# Risk of acute myocardial infarction with nonselective non-steroidal anti-inflammatory drugs: a meta-analysis

**DOI:** 10.1186/ar2047

**Published:** 2006-09-22

**Authors:** Gurkirpal Singh, Olivia Wu, Peter Langhorne, Rajan Madhok

**Affiliations:** 1 Division of Gastroenterology and Hepatology, Stanford University School of Medicine, 100 Hamilton Avenue, Suite 225 #42, Palo Alto, CA, 94301, USA; 2 Institute of Clinical Outcomes Research and Education, 100 Hamilton Avenue, Suite 225 #42, Palo Alto, CA, 94301, USA; 3 Division of Developmental Medicine, University of Glasgow, Glasgow Royal Infirmary, Castle Street, Glasgow, G4 0SF, UK; 4 Division of Cardiovascular Medicine and Medical Sciences, University of Glasgow, Glasgow Royal Infirmary, Castle Street, Glasgow, G4 0SF, UK; 5 Centre for Rheumatic Diseases, Glasgow Royal Infirmary, Castle Street, Glasgow, G4 0SF, UK

## Abstract

The use of cyclo-oxygenase 2 selective nonsteroidal anti-inflammatory drugs (NSAIDs) is associated with increased risk of acute myocardial infarction (AMI). The association between the risks of AMI with nonselective NSAIDs is less clear. We reviewed the published evidence and assessed the risk of AMI with nonselective NSAIDs. We performed a meta-analysis of all studies containing data from population databases that compared the risk of AMI in NSAID users with that in non-users or remote NSAID users. The primary outcome was objectively confirmed AMI. Fourteen studies met predefined criteria for inclusion in the meta-analysis. Nonselective NSAIDs as a class was associated with increased AMI risk (relative AMI risk 1.19, 95% confidence interval [CI] 1.08 to 1.31). Similar findings were found with diclofenac (relative AMI risk 1.38, 95% CI 1.22–1.57) and ibuprofen (relative AMI risk 1.11, 95% CI 1.06 to 1.17). However, this effect was not observed with naproxen (relative AMI risk 0.99, 95% CI 0.88–1.11). In conclusion, based on current evidence, there is a general direction of effect, which suggests that at least some nonselective NSAIDs increase AMI risk. Analysis based on the limited data available for individual NSAIDs, including diclofenac and ibuprofen, supported this finding; however, this was not the case for naproxen. Nonselective NSAIDs are frequently prescribed, and so further investigation into the risk of AMI is warranted because the potential for harm can be substantial.

## Introduction

One of the most revelatory issues concerning pharmaceuticals in recent years has been the relationship found between selective cyclo-oxygenase (COX)-2 inhibitors and cardiovascular thrombotic adverse events such as acute myocardial infarction (AMI) [1-5]. Received wisdom has never implicated the older class of similarly acting drugs, the nonsteroidal anti-inflammatory drugs (NSAIDs), in this association. However, new evidence suggests that there be an association between these nonselective NSAIDs and cardiovascular adverse effects, and that the risk may be similar with this class of drugs to that with COX-2 selective NSAIDs [6].

NSAIDs are among the most popular of prescribed drugs and have proven effectiveness in relieving symptoms of inflammation, including pain; they may also have a role in cancer prevention [7]. Both their benefits and adverse effects are due to the inhibition of either COX-1 or COX-2 enzymes. NSAIDs inhibit both COX-1 and COX-2, with extent of inhibition of COX-1 versus COX-2 differing between NSAIDs [8]. It is believed that the NSAID-induced inhibition of COX-1 in the gastrointestinal mucosa leads to the development of serious gastrointestinal complications such as ulcers and bleeds. The selective COX-2 inhibitors were developed to inhibit preferentially the COX-2 enzyme while sparing COX-1, with the premise that this would prevent serious gastrointestinal toxicity. However, recent studies have shown an unequivocal increase in risk of cardiovascular thrombotic events in patients treated with these drugs [1-5].

It is unclear whether the greater risk of AMI seen with selective COX-2 inhibitors is a 'class' effect of all NSAIDs. Like aspirin, nonselective NSAIDs inhibit COX-1, albeit temporarily, and they have generally been assumed to be antithrombotic or to have no cardiovascular adverse effect [8]. More recent studies suggest that many NSAIDs, both selective and nonselective, may result in an excess of AMIs [6]. In view of the large numbers of patients prescribed nonselective NSAIDs, we reviewed the available evidence and report the results of a meta-analysis conducted to determine whether nonselective NSAIDs increase AMI risk.

## Materials and methods

### Search strategy

We searched all major electronic databases including Medline, BIDS and EMBASE, between January 1980 and June 2005. Relevant keywords (as MeSH terms and text words) relating to NSAIDs (for instance, anti-inflammatory agents, non steroidal anti-inflammatory) and AMI (for instance, myocardial infarction, myocardial ischemia, cardiac ischemia, death) were combined to capture all potentially relevant studies. In addition, we contacted experts in the area and reviewed relevant discussions of the US Food and Drug Administration advisory panels and the UK National Institute of Clinical Excellence. Hand searching the reference lists of all relevant papers and recent topic reviews was also carried out.

### Study selection

Two reviewers assessed the studies retrieved from the search independently by scanning all the titles and abstracts. Full text copies of the selected papers were obtained and scrutinised independently by both reviewers for inclusion. Studies were included if they met the following criteria. The design of included studies was required to be observational studies of data from population databases that included comparison of NSAID use and non-use or remote-NSAID use. The intervention has to be use of nonselective NSAIDs. Comparison groups were required to be current NSAID users along with non-users or remote users. Finally, the outcome was required to be objectively confirmed AMI.

### Data extraction and quality assessment

Data from studies meeting the inclusion criteria were extracted into standardized data extraction forms independently by two reviewers (GS and RM), during which the quality of the studies was also assessed. This systematic review included a variety of study types. In order to maintain consistency of reporting, a validated generic checklist designed for evaluation of quantitative studies was used to assess the quality of all of the studies included in the review [9]. This checklist originally included 14 criteria, but one of these referred to random allocation of treatment and another referred to the blinding of participants. These were considered not applicable to observational studies and were excluded from the checklist; therefore, the final checklist consisted of 12 items. These items are consistent with the recommendations from the Centre for Reviews and Dissemination [10] and the consensus statement on meta-analysis reporting of observational studies in epidemiology [11]. Any disagreement relating to inclusion of studies, data extraction, or quality assessment between the reviewers was resolved by discussion.

### Data synthesis

We performed a meta-analysis using the random effects model based on the generic inverse variance method on nonselective NSAIDs as a class, and where data were available meta-analyses on individual NSAIDs were also carried out. The random effects model accounts for interstudy variance and provides a more conservative estimate of effect than does the fixed effect model, whereas the generic variance method can take into account confounding by combining adjusted relative risk estimates [12]. A pooled generic measure of relative AMI risk was calculated from the individual studies' estimates of relative AMI risk (expressed in relative risks and odds ratios) and 95% confidence intervals (CIs). We used the assumption that when the outcome of interest is rare, odds ratio approximates the relative risk. Potential sources of heterogeneity were investigated and assessed using the standard χ^2 ^test. In addition, the I^2 ^statistic was used to evaluate inconsistencies in results reported among the studies. In addition, we assessed publication bias graphically by using a funnel plot. Sensitivity analysis was carried out to assess the robustness of the results of the meta-analysis and to explore heterogeneity. All analyses were performed using RevMan 4.2 (Cochrane Collaboration, Oxford, UK).

## Results

**Figure 1:**
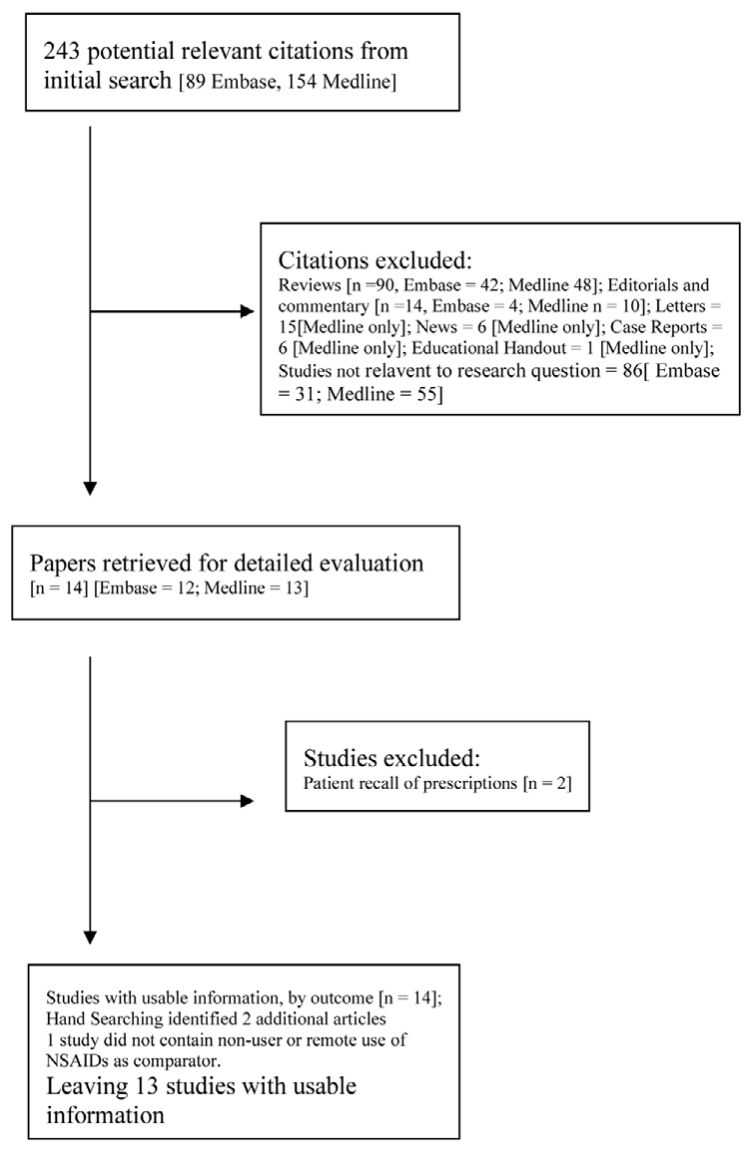
Selection of studies included in the meta-analysis.

The search strategy identified 243 potentially relevant citations for review (Figure 1). On reviewing retrieved papers, two were based on patient recall and thus were likely to be subject to reverse recall bias and were excluded [13,14]. A further two studies were identified by hand searching [15,16], of which one was excluded because it compared the effects of one NSAID with current use of three other NSAIDs but it did not have a control population of remote users or non-users [15].

Fourteen studies contained relevant data and were included in the analysis [4,16-28], and their characteristics are summarized in Table 1. All of the studies were based on data from validated databases from Canada, Denmark, the UK, and the USA. Most studies provided details on AMI risk with nonselective NSAIDs as a group, but some included information only on non-naproxen NSAIDs. Five studies provided information on dose: in one [19] the effect of dose was examined only in long-term users (no dose effect was seen); two studies [18,28] reported no effect of dose; one [27] reported greater risk of AMI with higher doses; and one study [24] reported a dose-dependent effect of ibuprofen but no effect with other NSAIDs. The duration of exposure in most studies was short (Table 1). Contrary to the majority of the studies included in the review, two studies were conducted in selected populations; one study [17] included only postmenopausal women and the other [16] was limited to patients with rheumatoid arthritis. In only three studies was the indication for prescription examined in detail [16,17,19].

**Figure 2:**
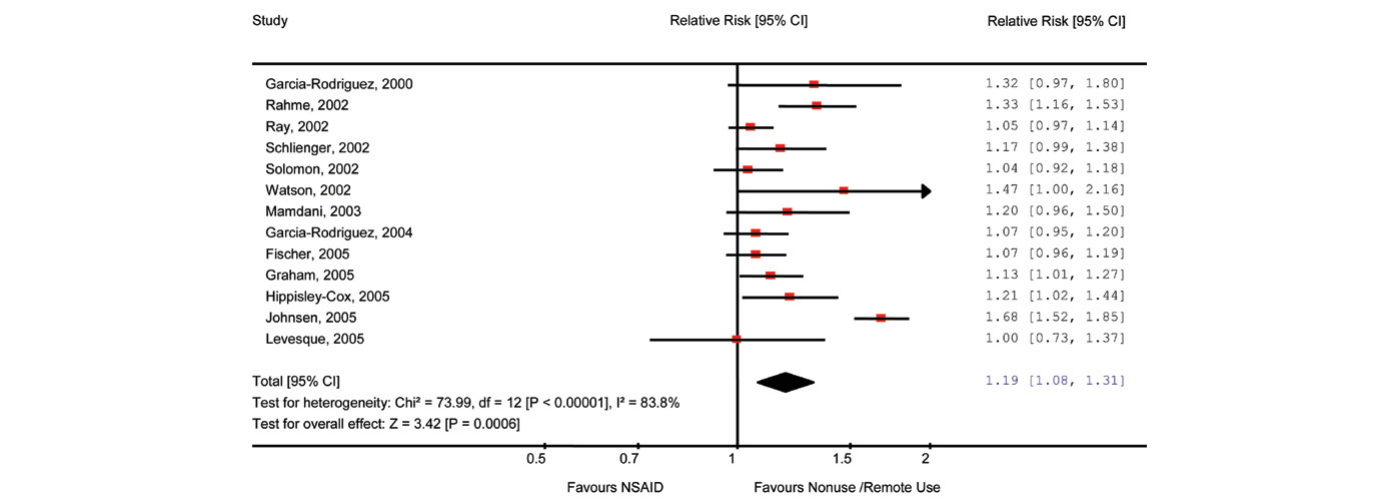
The relative AMI risk associated with use of nonselective NSAIDs versus remote use or non-use. There may be small discrepancies between the individual study values presented here and those presented in the original studies. This is due to the function of the software used for the calculation. One study did not present data on polled NSAIDs and is excluded from this figure. AMI, acute myocardial infarction; NSAID, nonsteroidal anti-inflammatory drug.

All of the studies were included in the meta-analysis (Figure 2). With the exception of one study [22], which reported no effect of nonselective NSAID use and AMI risk, all studies identified a similar trend toward increased risk of AMI. The meta-analysis of nonselective NSAIDs as a class was based on 13 studies [6,16-23,25-28], because one study presented data only on specific nonselective NSAIDs as part of an evaluation of the risks of AMI with COX-2 inhibitors [24]. The results revealed an AMI risk of 1.19 (95% CI 1.08 to 1.31; *P *= 0.0006) in NSAID users when compared with non-users or remote users. However, there was high between-study heterogeneity (I^2 ^= 83.8%; *P *< 0.00001).

Limited data were available on individual NSAIDs (Table 2). Five studies presented data on diclofenac [6,16,18,19,27] and reported increased AMI risk with diclofenac use compared with non-use or remote use of NSAIDs; the pooled relative AMI risk was 1.38 (95% CI 1.22 to 1.57; *P *< 0.00001). There was no significant between-study heterogeneity (I^2 ^= 54%; *P *= 0.08). Nine studies evaluated the association between ibuprofen and the risk of AMI [6,16,18-20,24,25,27,28]. Although the majority of the individual studies reported nonsignificant risk association, the pooled analysis identified a relative AMI risk of 1.11 (95% CI 1.06 to 1.17; *P *= 0.0001). No evidence of heterogeneity was detected (*P *= 0.41), and the risk estimates of individual studies were consistent (I^2 ^= 3.2%). In contrast, naproxen use was not found to be associated with increased AMI risk (relative AMI risk 0.99, 95% CI 0.88 to 1.11; *P *= 0.99). The studies included in the analysis yielded conflicting results. Eight studies [16,18,19,23-25,27,28] indicated that naproxen was not associated with increased AMI risk, whereas four studies [6,20-22] suggested that naproxen was associated with increased risk of AMI. The risk estimated in two of the latter four studies [6,21] was statistically significant. Overall, significant heterogeneity (*P *= 0.01) and moderate inconsistency (I^2 ^= 54%) were present among the estimates reported by the studies.

### Sensitivity analysis

Sensitivity analysis was carried out to explore the heterogeneity and inconsistencies of the results of the studies included in the meta-analysis. We first analyzed the nonselective NSAID data by excluding the two studies based on selected populations [15,25]; this had little impact on the results of the meta-analysis (relative AMI risk 1.18, 95% CI 1.05 to 1.33; *P *= 0.005), and significant heterogeneity remained (*P *< 0.00001). One of the studies [21] exhibited a higher than expected risk of AMI compared with the other studies; the analysis was therefore repeated with this study's data excluded. The results showed a relative AMI risk of 1.13 (95% CI 1.07–1.18; *P *< 0.00001); there was significant heterogeneity (*P *= 015), and a small-to-moderate degree of inconsistency remained (I^2 ^= 30.7%). All of the analyses were also repeated using a fixed effect model, but there was little change in the results.

## Discussion

Considerable scientific and media attention has been directed at reports that selective COX-2 inhibitors increase AMI risk. Two selective COX-2 inhibitors have been withdrawn, and the sales of another have plummeted. However, until recently, little attention has been focused on the risks associated with use of the nonselective NSAIDs. Because there is no randomised controlled study of nonselective NSAIDs large enough to detect an increase in a common condition such as AMI, an absence of evidence has been assumed to imply evidence of absence. Our meta-analysis shows that use of at least some nonselective NSAIDs is associated with a small but significantly increased risk of AMI compared with remote and non-use. If this small increase is indeed causally related to use of nonselective NSAIDs, then the implications for public health policy are considerable because of the large numbers of patients prescribed these drugs.

We also investigated the relative AMI risk associated with frequently prescribed nonselective NSAIDs, including diclofenac, ibuprofen, and naproxen, individually. When comparing the use of diclofenac and ibuprofen with no or remote NSAID use, the results supported the presence of increased risk of AMI, similar to that observed with NSAIDs as a class. We did not find a significant association between naproxen use and AMI, but there was significant heterogeneity and moderate inconsistency among the 12 studies. It is possible that our meta-analysis was confounded by pharmaceutical company support of several naproxen studies in the wake of the increased risk of AMI seen with rofecoxib in the VIGOR (VIOXX GI Outcomes Research) study [29,30]. The increased risk of AMI observed with rofecoxib in the VIGOR trial was explained by a purported 'cardioprotective' effect of naproxen. Several epidemiologic studies were funded by the manufacturer of rofecoxib to prove the cardioprotective effect of naproxen. These studies, included in our meta-analysis, indicate that there was a large cardioprotective effect of naproxen, unlike most other independently funded studies. This largely explains the between-study heterogeneity in our analysis, as previously established by Juni and coworkers [29]. An interim analysis of an Alzheimer's disease prevention study [31] has suggested increased risk of AMI in patients treated with naproxen, but the full data have not yet been released.

It is possible that different NSAIDs may be associated with differential increases in risk of AMI. Differences between NSAIDs, based on their pharmacologic effects, have been previously described for the risk of gastrointestinal bleeding [32]. However, there are few data available in the literature on the cardiovascular risk of NSAIDs other than ibuprofen, diclofenac, and naproxen.

There are several potential limitations to our study, many of which are inherent to all meta-analysis of observational studies. The quality of our analysis depends on the data extracted from the original publications; we may thus inherit the problems of potential bias and confounding by indication inherent to observational studies. It is possible that sicker patients may preferentially receive NSAID treatment, and these patients carry a higher baseline risk of cardiovascular complications (confounding by indication). All of the observational studies included in our meta-analysis adjusted for this confounding, but it is possible that this limitation was not completely eliminated because of unmeasured variables. However, one advantage of observational studies is that they more accurately reflect the spectrum of patients in clinical practice, and if they are large enough they can detect rare adverse events or increased occurrence of a common disorder [33]. Randomized controlled trials are more appropriate for defining efficacy and assigning causality, but their external validity or generalizability can often be low [34] and they are rarely sufficiently large or long running to identify all adverse events [35,36]. Although the randomized clinical trial remains the 'gold standard', it is unlikely that a large clinical trial to study the effect of all NSAIDs on cardiovascular risk will ever be conducted, thus emphasizing the need for critical evaluation of observational studies.

The statistical pooling of risk from observational studies is controversial because of the many biases that can arise in observational studies compared with randomized controlled trials. It has been argued that presenting a single pooled estimate without additional detail can be misleading but is justified under certain circumstances [37]. In our study the differences in risk estimates between the individual studies was small, and study designs were similar, justifying the pooling of the studies. A formal test of heterogeneity in our overall analysis showed that chance was not the explanation. In an attempt to explain this we undertook a *post hoc *investigation by study type and study population. One study with a much higher than expected AMI risk [21] was found to be contributing to the heterogeneity; the reasons for this was not apparent. It is possible that some of the heterogeneity may reflect the relative proportions of different NSAIDs used in the study populations.

None of the studies sought information on use of nonselective NSAIDs purchased over the counter; it is thus not possible to exclude bias arising from their use. Five of the studies were from the same database [16-19,27]. Two of the studies were restricted to either rheumatoid arthritis patients or postmenopausal women [16,17]. However, the exclusion of these studies did not change the pooled estimate of AMI risk. The three other studies [18,19,27] were undertaken over separate 5-year intervals and recorded first episode AMIs occurring during the study interval. It is thus unlikely that their inclusion altered the pooled risk estimate because of duplication. To further examine whether the inclusion of all studies was biasing the results, the analysis was repeated with the study with lowest AMI risk [18], and there was no difference in overall risk.

Another potential concern is that some of the studies relied on pharmaceutical company sponsorship and thus need to be interpreted with caution; this is particularly relevant in relation to naproxen. Furthermore, we did not include meeting abstracts in our analysis because frequently insufficient information could be extracted.

The results of our meta-analysis are largely consistent with observations from studies of selective COX-2 inhibitors. In most observational studies of COX-2 inhibitors the estimated relative risk of AMI ranged between 0.8 and 1.5, similar to the risk we found with nonselective NSAIDs [31]. Large randomized controlled trials comparing COX-2 inhibitors with nonselective NSAIDs have not invariably found an increased risk of AMI. In the VIGOR study rofecoxib 50 mg had a relative risk of 2 compared with naproxen for the composite end-point of death, stroke, and AMI [30]. Celecoxib, when compared with ibuprofen and naproxen, was not associated with any difference in the number of severe adverse cardiovascular events in the CLASS (Celecoxib Long-term Arthritis Safety Study) study [38]. Similarly, in the EDGE (Etoricoxib Diclofenac Gastrointestinal Evaluation) study [39], which compared etoricoxib with diclofenac in patients with osteoarthritis, there was no overall difference between the two drugs in serious adverse cardiovascular events. With regard to lumiracoxib, in the TARGET (Therapeutic Arthritis Research and Gastrointestinal Event Trial) study [40] the primary analysis revealed no difference when naproxen and ibuprofen were considered as a single group. In a substudy analysis lumiracoxib carried a greater risk than naproxen but less than that of ibuprofen. It has therefore been suggested cardiovascular events, including AMIs, may arise as class effect of both COX-2 selective and nonselective NSAIDS. This has resulted in the US Food and Drug Adminiatration advising manufacturers that a boxed warning of an increased risk of serious cardiovascular adverse events should accompany all NSAIDs, including those available over the counter [31].

The role of concomitant aspirin in altering the risk of thromboembloic complications with COX-2 inhibitors is controversial [31]. Although not a primary aim of our study, four studies [6,18,19,22] did report on concomitant aspirin use with NSAIDs; three found no effect and one a further reduction in events, but these observations were based on a small number of events. Larger studies are needed to study the effect of concomitant aspirin use. Similarly, there is inadequate data on the effect of dose and duration of use of NSAIDs.

The occurrence of AMI with NSAID could arise because of a number of the consequences of COX inhibition. The most frequently proposed theory regarding the excess of AMI with selective COX-2 inhibitors is one of thromboxane/prostacyclin imbalance [41,42]. Grosser and coworkers [42] postulated that nonselective NSAIDs would also increase the risk of AMI, and that this increase in risk would be dependent on the COX-2 selectivity of the NSAID, with drugs such as diclofenac carrying higher risk than naproxen; the results of our study are consistent with this. NSAID-induced hypertension is also well recognized, and even small sustained increases in blood pressure can significantly increase the risk of adverse cardiovascular events [42,43]. One estimate projects 35,700 additional events per annum from use of NSAIDS in rheumatoid arthritis and osteoarthritis patients alone [44].

## Conclusion

Acknowledging the limitations of observational data, our systematic review of the only available published data indicates that several nonselective NSAIDs are associated with increased risk of AMI. Until results become available from a randomized controlled trial large enough to detect the risk we found (which, we consider, is unlikely ever to be undertaken), we endorse the pragmatic advice that the lowest possible dose should be used for the shortest possible duration [45] for all NSAIDs. Furthermore, we add that these drugs, like COX-2 inhibitors, should be used with caution in those with risk factors for atheromatous vascular disease and should be avoided in those with clinical complications. Patient selection rather than drug selectivity may thus be more important in their use.

## Abbreviations

AMI = acute myocardial infarction; CI = confidence intervals; COX = cyclo-oxygenase; NSAID = nonsteroidal anti-inflammatory drug.

## Competing interests

Institute of Clinical Outcomes Research and Education has received research grants from Boehringer-Ingelheim, Glaxo Smith Kline, Novartis and Pfizer, and GS has been a speaker for Pfizer. RM has received ducational grants from Abbott Laboratories, Strakan Ltd., and Merck. OW and PL declare that they have no competing interests. There was no pharmaceutical company funding in support of this study.

## Authors' contributions

RM conceived the study. GS, OW, and RM collected and analyzed the data. PL advised on data analysis. All authors were involved in writing the report and approved the final manuscript.

## Figures and Tables

**Table 1:** Characteristics of included studies

Ref.	Design	Data source	NSAID exposure	Study outcome	AMI events (*n*)	No AMI events (*n*)	Confounding factors
[19]	Nested case control	GPRD (UK)	Current NSAID users: patients (age 50–84 years) whose most recent prescription lasted until the index date or ended in the 30 days before the index date	First hospitalized AMI or death due to CHD	Diclofenac = 213 Ibuprofen = 155 Naproxen = 49	Diclofenac = 679 Ibuprofen = 575 Naproxen = 206	MI risk factors (smoking, diabetes, hypertension, hyperlipidemia, BMI, RA, OA, anemia, CHD, CVD), age, sex, calendar year, alcohol intake, and use of steroids, aspirin, anticoagulants, paracetamol, and NSAIDs
[17]	Nested case control	GPRD (UK)	Current NSAID users: menopausal women (age 50–74 years) who had a prescription for NSAIDs before the index date	First hospitalized AMI	Nonaspirin NSAIDs = 40	Nonaspirin NSAIDs = 143	Age, use of HRT, smoking, hypertension, diabetes, obesity, surgical menopause, family history of CHD, and predefined co-morbidity
[20]	Nested case control	Managed care database (USA)	Current NSAID users: patients (age 18–84 years) who had ≥1 prescription for a COX 2 selective or non-selective NSAID	Hospitalized AMI, sudden cardiac death	Ibuprofen = 670 Naproxen = 367	Ibuprofen = 2573 Naproxen = 1409	Sex, age, geographic location, cardiovascular risk score, admission for noncardiac-related disorders and same-day procedures, emergency room visits for noncardiovascular reasons, HRT, and high-dose prednisolone
[6]	Nested case control	QRESEARCH (UK)	Current NSAID users: patients (age 25–100 years) who had a prescription for selective or nonselective NSAIDs within the 3 months before the index data	First hospitalized AMI/CHD, sudden death	Ibuprofen = 460 Naproxen = 96 Other nonselective NSAIDs = 181	Ibuprofen = 3199 Naproxen = 677 Other nonselective NSAIDs = 1266	Use of aspirin, statin, tricyclic antidepressants, SSRI, ischemic heart disease, diabetes, hypertension, OA, RA, smoking obesity, and deprivation
[21]	Case control	Hospital discharge registry (Denmark)	Current nonaspirin NSAID users: patients (age 20–101 years) who had received a prescription within 30 days before the index date	First hospitalized AMI	Naproxen = 26 Other nonaspirin NSAIDs = 532	Naproxen = 175 Other nonaspirin NSAIDs = 3105	Discharge diagnosis of CVD, hypertension, diabetes, chronic bronchitis or emphysema, acholoism, liver cirrhosis, upper GI bleed, RA, systemic lupus erythematosus and use of high-dose aspirin, platelet inhibitors, insulin or oral hypoglycemic drugs, antihypertensive drugs, lipid-lowering drugs, oral anticoagulants, hormone therapy, nitrates, penicillamine, gold and glucocorticocoids before date of admission
[22]	Nested case control	Administrative health database (Québec, Canada)	New NSAID users: patients (age ≥66 years) who had a dispensed prescription with a duration that covered or overlapped with the index date	First Hospitalized AMI	Naproxen = 23 Other nonselective, nonaspirin NSAIDs = 51	Naproxen = 336 Other nonselective, nonaspirin NSAIDs = 962	Age, sex, hypertension, CAD, cerebrovascular disease, peripheral vascular disease, CCF, statin, aspirin, anticoagulants, presence of respiratory disease, GI ulcer disease, thyroid disease, depression or psychiatric illness, use of oral corticosteroids, Chronic disease score, Charlson index, health care utilization
[23]	Retrospective cohort	Administrative health database (Ontario, Canada)	New NSAID users: patients (age ≥66 years) who received a prescription for NSAIDs	Hospitalized AMI	Naproxen = 15 Other nonselective, non-naproxen NSAIDs = 134	Naproxen = 5654 Other nonselective, nonaspirin NSAIDs = 33,734	Hospitalization in prior year, malignancy in prior 5 years, MI, stroke, CAD or CABG in prior 5 years, age, sex, long-term care, low income, number of different drugs
[24]	Retrospective cohort	Tennessee Medicaid (USA)	New nonaspirin NSAID users: patients (age 50–84 years) who had a prescription of NSAIDs, with no use during the previous 365 days	Hospitalized AMI or death from CHD	Ibuprofen = 339 Naproxen = 201 Other or multiple nonaspirin NSAIDs = 301	Data not available	Prescribed drugs for CVS disease, hospital admissions and emergency visits for CVS and other disease, PVD, CVD, CAD, and revascularization procedures
[25]	Retrospective cohort	Tennessee Medicaid (USA)	Current and new NSAID users: patients (age 50–84 years) who were taking NSAIDs at enrolment were classed as current users; those who began use of an NSAID during the follow-up period were classed as new users	Hospitalized AMI or death from CHD	Current users: Ibuprofen = 190 Naproxen = 245 New users: Ibuprofen = 52 Naproxen = 72	Data not available	Prescribed drugs for CVS disease, hospital admissions and emergency visits for CVS and other disease, PVD, CVD, CAD, and revascularization procedures
[26]	Matched case control	Administrative health database (Québec, Canada)	Current NSAID users: patients (age ≥65 years) who had a dispensed prescription with a duration that covered or overlapped with the index date	First AMI	Naproxen = 255 Other NSAIDs = 1062	Naproxen = 212 Other NSAIDs = 722	Prior use of anticoagulants, nitrates, lipid lowering agents, antidiabetic agents, antihypertensive agents, prior CVD, presence of co-morbidity factors
[27]	Nested case control	GPRD (UK)	Current NSAID users: patients (age ≤75 years) who had their last prescription for an NSAID before the index date and which ended at or after the index date	First hospitalized AMI	Diclofenac = 97 Ibuprofen = 60 Naproxen = 19	Diclofenac = 277 Ibuprofen = 204 Naproxen = 105	Aspirin, BMI, smoking, HRT
[28]	Case control	Medicaid/Medicare (New Jersey, USA)	NSAID users: patients (age ≥60 years) who had use of prescribed NSAIDs during the 6 months before the index date	Hospitalized AMI	Ibuprofen = 285 Naproxen = 243	Ibuprofen = 1030 Naproxen = 1094	Hypertension, diabetes, CCF, and validated co-morbidity index
[16]	Case control	GPRD (UK)	Current NSAID users: patients (age 40–79 years) with RA and who had received a NSAID prescription during the 30 days before the index date	First AMI, sudden death and stroke	Data not available	Data not available	Adjusted values reported, but factors that were adjusted for were not detailed
[18]	Case control	GPRD (UK)	Current NSAID user: patients <89 years whose prescription overlapped with the index date	First AMI	Nonaspirin NSAID = 680 Diclofenac = 260 Ibuprofen = 176 Naproxen = 63	Nonaspirin NSAID = 2339 Diclofenac = 834 Ibuprofen = 656 Naproxen = 251	Adjusted for hypertension, hyperlipidemia, diabetes, ischemic heart disease, BMI, kidney disease, RA, and aspirin use

AMI, acute myocardial infarction; BMI, body mass index; CABG, coronary artery bypass grafting; CAD, coronary artery disease; CCF, congestive cardiac failure; CHD, coronary heart disease; COX, cyclo-oxygenase; CVD, cardiovascular disease; CVS, cardiovascular system; GI, gastrointestinal; GPRD, General Practice Research Database; HRT, hormone replacement therapy; MI, myocardial infarction; NSAID, nonsteroidal anti-inflammatory drug; OA, osteoarthritis; PVD, peripheral vascular disease; RA, rheumatoid arthritis; SSRI, selective serotonin reuptake inhibitor.

**Table 2:** The relative AMI risk associated with the use individual NSAIDs compared with remote or no use.

Drug [ref.]	Relative AMI risk	95% CI	Test for heterogeneity		Test for inconsistency
			
			χ^2^	*P *value	I^2^
Diclofenac	1.41	1.20–1.65	9.79	0.08	48.9%
[27]	1.38	1.08–1.77			
[16]	1.68	1.14–2.48			
[19]	1.18	0.99–1.40			
[6]	1.55	1.39–1.72			
[18]	1.23	1.00–1.51			
Ibuprofen	1.11	1.04–1.18	8.26	0.41	3.2%
[25]	1.15	1.03–1.29			
[24]	1.01	0.77–1.33			
[27]	1.17	0.87–1.58			
[28]	1.02	0.88–1.18			
[16]	0.74	0.35–1.56			
[19]	1.06	0.87–1.29			
[20]	1.06	0.96–1.17			
[6]	1.24	1.11–1.39			
[18]	1.16	0.92–1.46			
Naproxen	0.99	0.88–1.12	23.91	0.01	54%
[25]	0.95	0.82–1.10			
[24]	0.93	0.74–1.17			
[27]	0.68	0.41–1.12			
[28]	0.84	0.72–0.98			
[16]	0.57	0.31–1.05			
[23]	1.00	0.59–1.68			
[19]	0.89	0.64–1.24			
[20]	1.14	1.00–1.30			
[6]	1.27	1.01–1.60			
[21]	1.50	0.99–2.28			
[22]	1.17	0.75–1.83			
[18]	0.96	0.66–1.39			

AMI, acute myocardial infarction; CI, confidence interval; NSAID, nonsteroidal anti-inflammatory drug.
